# P-276. Outcomes Following Cefiderocol vs. Meropenem Treatment for Gram-Negative Pneumonia: A Real-World Data Analysis

**DOI:** 10.1093/ofid/ofae631.480

**Published:** 2025-01-29

**Authors:** Benjamin S Haslund-Gourley

**Affiliations:** Drexel College of Medicine, San Ramon, California

## Abstract

**Background:**

The prevalence of multidrug-resistant (MDR) gram-negative pneumonia continues to increase across the United States (U.S.). Recently, cefiderocol (Fetroja), was approved to treat MDR-negative pneumonia, overcoming several beta-lactam resistance mechanisms. While cefiderocol has been utilized over the last 6 years, additional studies are needed to assess its use long-term. The purpose of this study was to compare outcomes of gram-negative pneumonia patients treated with either cefiderocol or meropenem utilizing real-world data.

**Methods:**

This was a retrospective cross-sectional study between 05/02/2018 and 05/02/2024 utilizing the TriNetX Research Network, consisting of 88 U.S. and ex-U.S. healthcare organizations. Patients were included if they had an ICD-10 code for gram-negative pneumonia and receipt of cefiderocol or meropenem after pneumonia diagnosis, defined by RxNorm codes. Antibiotic receipt after pneumonia diagnosis defined the index event date. Patient cohorts were balanced using demographic (age, sex, race) and clinical data (antibiotic use, and XX co-morbidities) via a 1:1 greedy, nearest neighbor propensity score matching (PSM) with a 0.1 caliper. Outcomes following this index event included mortality, *Clostridioides difficile* infection (CDI), epilepsy/recurrent seizures, coagulation defects, pulmonary fibrosis (PF), and sepsis. A Kaplan-Meier Analysis was used to compare the proportion of ceficerocol or meropenem patients with each outcome of interest one-year post-index.

**Results:**

After PSM, cefiderocol (n=94) showed no significant differences in mortality (HR 1.38, 95% 0.87-2.18), CDI (HR 1.04, 95% 0.26-4.17), epilepsy/recurrent seizures (HR 1.64, 95% 0.67-4.01), PF (HR 1.20, 95% 0.40-3.60), or sepsis (HR 1.17, 95% 0.75-1.81) one year post-index vs. meropenem (n=94). Cefiderocol patients had lower rates of coagulation defects (HR 0.2, 95% 0.04-0.91, p=0.021).

**Conclusion:**

Overall, cefiderocol patients showed no differences in mortality, CDI, epilepsy/recurrent seizures, PF, or sepsis. However, patients treated with cefiderocol for gram-negative pneumonia had a lower rate of coagulopathies. Additional real-world studies are needed to monitor its long-term outcomes.

**Disclosures:**

**All Authors**: No reported disclosures

